# A DNS Study of Closure Relations for Convection Flux Term in Transport Equation for Mean Reaction Rate in Turbulent Flow

**DOI:** 10.1007/s10494-017-9833-y

**Published:** 2017-08-01

**Authors:** A. N. Lipatnikov, V. A. Sabelnikov, N. Chakraborty, S. Nishiki, T. Hasegawa

**Affiliations:** 1 Department of Applied Mechanics, Chalmers University of Technology, 412 96 Gothenburg, Sweden; 2 ONERA – The French Aerospace Laboratory, 91761 Palaiseau, France; 3 Central Aerohydrodynamic Institute (TsAGI), 140180 Zhukovsky, Moscow Region Russian Federation; 4 School of Mechanical and Systems Engineering, Newcastle University, Newcastle Upon Tyne, NE1 7RU UK; 5 Department of Mechanical Engineering, Kagoshima University, Kagoshima, 890-0065 Japan; 6 Institute of Materials and Systems for Sustainability, Nagoya University, Nagoya, 464-8603 Japan

**Keywords:** Premixed turbulent combustion, Modeling, Turbulent flux, Countergradient transport, DNS

## Abstract

The present work aims at modeling the entire convection flux $\overline {\rho \mathbf {u}W}$ in the transport equation for a mean reaction rate $\overline {\rho W}$ in a turbulent flow, which (equation) was recently put forward by the present authors. In order to model the flux, several simple closure relations are developed by introducing flow velocity conditioned to reaction zone and interpolating this velocity between two limit expressions suggested for the leading and trailing edges of the mean flame brush. Subsequently, the proposed simple closure relations for $\overline {\rho \mathbf {u}W}$ are assessed by processing two sets of data obtained in earlier 3D Direct Numerical Simulation (DNS) studies of adiabatic, statistically planar, turbulent, premixed, single-step-chemistry flames characterized by unity Lewis number. One dataset consists of three cases characterized by different density ratios and is associated with the flamelet regime of premixed turbulent combustion. Another dataset consists of four cases characterized by different low Damköhler and large Karlovitz numbers. Accordingly, this dataset is associated with the thin reaction zone regime of premixed turbulent combustion. Under conditions of the former DNS, difference in the entire, $\overline {\rho {u}W}$, and mean, $\tilde {u}\overline {\rho W}$, convection fluxes is well pronounced, with the turbulent flux, $\overline {\rho u^{\prime \prime }W^{\prime \prime }}$, showing countergradient behavior in a large part of the mean flame brush. Accordingly, the gradient diffusion closure of the turbulent flux is not valid under such conditions, but some proposed simple closure relations allow us to predict the entire flux $\overline {\rho \mathbf {u}W}$ reasonably well. Under conditions of the latter DNS, the difference in the entire and mean convection fluxes is less pronounced, with the aforementioned simple closure relations still resulting in sufficiently good agreement with the DNS data.

## Introduction

The critical point of the turbulent combustion theory stems from averaging reaction rates subject to fluctuations in the local temperature *T* and concentrations. This is an issue of severe importance, because (i) the rates of reactions that control heat release depend on *T* in a highly non-linear manner, (ii) the magnitudes of the temperature fluctuations are typically large in turbulent flames, and (iii) these fluctuations exhibit a wide range of length and time scales. As reviewed elsewhere [1–7], among the most widely used approaches to solving this highly non-linear and multiscale problem, there are methodologies that deal with the Reynolds-averaged or filtered form of (i) the following transport equation

1 $$ \frac{\partial} {\partial t}\left( \rho c \right)+{\nabla} \cdot \left( \rho \mathrm{\mathbf{u}}c \right)={\nabla} \cdot \left( \rho D{\nabla} c \right)\mathrm{\mathbf{+}}\rho W $$

for a combustion progress variable *c*, which is assumed to completely characterize the state of a reacting mixture, and (ii) an extra transport equation for either mean scalar dissipation rate $\tilde {\chi } =\overline {D\nabla c\cdot \nabla c} $ [6, 8] or mean flame surface density $\bar {\Sigma } =\overline {\left | {\nabla } c \right |}$ [3, 4, 9–11], with the mean rate $\widetilde {W}$ being hypothesized to be linearly related to either $\tilde {\chi } $ or $\bar {\Sigma } $. More specifically, $\bar {\rho } \widetilde {W}$ is often considered to be equal to $\rho _{u}S_{L}\overline {\Sigma }$ [3, 4, 9–11] and the following linear relation $\widetilde {W}=\tilde {\chi } / \left (2c_{m}-1 \right )$ was derived by Bray [12] by assuming that the probability of finding intermediate (between unburned and fully burned) states of a reacting mixture is much less than unity. Here, *t* is time, **u** is the flow velocity vector, *ρ* is the mixture density, the combustion progress variable may be defined as follows ${c=\left (T-T_{u} \right )} / \left (T_{b}-T_{u} \right )$ in the simplest case of adiabatic burning of an equidiffusive mixture (i.e. *D*_*n*_ = *D* for all species *n* = 1,…,*N*) characterized by unity Lewis number (i.e. *L* *e* = *κ*/*D* = 1) and a low Mach number, *κ* is the heat diffusivity of the mixture, *W* is the rate of production of the combustion progress variable in the flame, *S*_*L*_ is the laminar flame speed, $c_{m}=\overline {\rho cW} / \overline {\rho W}$ is commonly assumed to be constant [6, 12], $\overline {q}$ and $\tilde {q}=\overline {\rho q} / \bar {\rho } $ are the Reynolds and Favre-averaged values of a quantity *q*, respectively, and subscripts *u* and *b* designate unburned and burned mixture, respectively.

It is worth noting, however, that even the precise knowledge of $\tilde {\chi } $ or $\bar {\Sigma } $ does not allow us to precisely evaluate $\widetilde {W}$, because the linear relations between these three quantities are just assumptions, which are best justified if unburned and fully burned gases are separated by a thin zone (flamelet) that retains the structure of the laminar flame. Nevertheless, recent analysis [13, 14] of Direct Numerical Simulation (DNS) data shows that, even under such conditions (i.e. in the flamelet regime of premixed turbulent combustion [1–8]), the linear relations do not hold in certain flame zones. For instance, a ratio of ${\bar {\rho } \widetilde {W}} / \left (\rho _{u}S_{L}\bar {\Sigma } \right )$ can be significantly larger than unity at $\overline {c}>0.8$ [14] and *c*_*m*_ strongly varies at $\overline {c}\ll 1$ [13] (it is worth remembering that the leading edge of a premixed turbulent flame brush may play a crucial role in the flame propagation, as discussed in detail elsewhere [7]). Accordingly, it would be of interest to straightforwardly evaluate the mean rate $\widetilde {W}$ by solving an appropriate transport equation without invoking extra assumptions regarding a relation between $\widetilde {W}$ and $\tilde {\chi } $ or $\bar {\Sigma } $.

This requirement is addressed in recent papers by the present authors [15, 16] where the following transport equations

2 $$ \frac{\partial} {\partial t}\left( \rho W \right)+{\nabla} \cdot \left( \rho \mathrm{\mathbf{u}}W \right)={\nabla} \cdot \left( \rho D{\nabla} W \right)\mathrm{\mathbf{-}}\rho \chi \frac{d^{2}W}{dc^{2}}\mathrm{\mathbf{+}}\rho W\frac{dW}{dc}, $$

3 $$ \frac{\partial} {\partial t}\left( \bar{\rho} \widetilde{W} \right)+{\nabla} \cdot \left( \bar{\rho} \tilde{\mathbf{u}}\widetilde{W} \right)+{\nabla} \cdot \overline{\rho \mathbf{u}^{\prime\prime}W^{\prime\prime}}={\nabla} \cdot \overline{\rho D\nabla W}\mathrm{\mathbf{-}}\overline{\rho \chi \frac{d^{2}W}{dc^{2}}}\mathrm{\mathbf{+}}\overline{\rho W\frac{dW}{dc}} $$

for the instantaneous and mean rates of product creation, i.e. *W* and $\widetilde {W}$ have been derived starting from Eq. 1 and assuming that *W* is solely controlled by *c*, i.e. $W=W\left (c \right )$. This assumption holds, in particular, in the case of adiabatic burning, low Mach number, single-step chemistry, equal diffusivities of fuel and oxidant, and *L* *e* = 1.0. The same simplifications are commonly invoked by models that deal with transport equations for $\tilde {\chi } $ [6, 8] or $\bar {\Sigma } $ [3, 4, 9–11]. Here, $q^{\prime \prime }=q-\tilde {q}$ for any quantity *q*. The statistical behaviors of the transport equations of $\widetilde {W}$ and $\bar {\Sigma } $ have been compared elsewhere [16] in the context of RANS simulations and the interested reader is referred to Ref. [16] for further information.

It is worth noting that (i) counterparts of Eq. 2 can be derived in more challenging cases (e.g., *L* *e* ≠ 1 or complex combustion chemistry) and (ii) Eq. 2 may also be filtered [16] for Large Eddy Simulation (LES) of turbulent flames. Nevertheless, before addressing more complicated problems, it is worth analyzing the newly introduced approach [15, 16] from the simplest case by thoroughly investigating the basic features of Eq. 3.

In order to apply Eq. 3 to simulations of premixed turbulent flames, closure relations for the third term on the Left Hand Side (LHS) and for the three terms on the Right Hand Side (RHS) should be developed. In this regard, the biggest challenge consists of the fact that the magnitudes of the second and third terms on the RHS are much higher than the magnitudes of other terms under typical conditions, whereas the signs of the two dominant terms are opposite [15, 16]. Accordingly, if separate closure relations are developed for each of the two dominant terms, even small uncertainties that stem from the closure relations can yield large residuals for Eq. 3. This challenging problem was resolved in Ref. [15], where the following relation

4 $$ {\nabla} \cdot \overline{\rho D\nabla W}\mathrm{\mathbf{-}}\overline{\rho \chi \frac{d^{2}W}{dc^{2}}}\mathrm{\mathbf{+}}\overline{\rho W\frac{dW}{dc}}=\bar{\rho} \widetilde{W}\left\langle {\dot{s}} | {c_{1}<c<c_{2}} \right\rangle $$

was proposed to jointly close the three terms on the RHS of Eq. 3. Then, Eq. 3 reads

5 $$ \frac{\partial} {\partial t}\left( \bar{\rho} \widetilde{W} \right)+{\nabla} \cdot \left( \bar{\rho} \tilde{\mathbf{u}}\widetilde{W} \right)+{\nabla} \cdot \overline{\rho \mathbf{u}^{\prime\prime}W^{\prime\prime}}=\bar{\rho} \widetilde{W}\left\langle {\dot{s}} |{c_{1}<c<c_{2}} \right\rangle $$

and Eq. 5 was validated [15, 16] with respect to the DNS data, which will be discussed later. Here, $\left \langle {\dot {s}}| {c_{1}<c<c_{2}} \right \rangle $ denotes stretch rate $\dot {s}={\nabla } \cdot \mathrm {\mathbf {u}}-\mathrm {\mathbf {nn}}:\mathrm {\nabla \mathbf {u}}+S_{d} {\nabla \cdot \mathbf {n}}$ conditioned to the reaction zone, which is bounded by *c*_1_ and *c*_2_ such that $\rho W\left (c_{1} \right )=\rho W\left (c_{2} \right )={\max \left \{ \rho W\left (c \right ) \right \}} / 2$, the unit vector $\mathrm {\mathbf {n=-}}{{\nabla } c} / \left | {\nabla } c \right |$ is locally normal to the instantaneous flame surface, and $S_{d}=\left [ {\nabla } \cdot (\rho D{\nabla } c)+\rho W \right ] / \left (\rho \left | {\nabla } c \right | \right )$ is the local displacement speed.

Nevertheless, two terms $\overline {\rho \mathbf {u}^{\prime \prime }W^{\prime \prime }}$ and $\left \langle {\dot {s}} | {c_{1}<c<c_{2}} \right \rangle $ should still be modeled in Eq. 5. As the discussed approach was put forward very recently [15, 16], the present communication is restricted to analyzing a single unclosed term in Eq. 5. This contributes to the ultimate goal to develop closure relations for the entire Eq. 3 in order to subsequently use it in RANS and LES studies of flames investigated in experiments. Thus the particular goal of the present work is to assess several simple closure relations for the former (turbulent flux) term by analyzing two sets of DNS data obtained from statistically planar flames associated with the flamelet and thin reaction zone regimes [1] of premixed turbulent combustion.

It is worth noting that assumptions invoked in the following to close the turbulent flux term are more clear if they are applied to the entire convection term $\overline {\rho \mathbf {u}W}$. Accordingly, in the rest of the paper, we will address closure relations for the entire convection term, whereas the counterpart closure relation for the turbulent transport term can be obtained using the following identity $\overline {\rho \mathbf {u}^{\prime \prime }W^{\prime \prime }}=\overline {\rho \mathbf {u}W}-\bar {\rho } \tilde {\mathbf {u}}\widetilde {W}$ and the closure relation for $\overline {\rho \mathbf {u}W}$. Such an approach is fully justified, because the final goal consists in evaluating the sum of the mean convection $\bar {\rho } \tilde {\mathbf {u}}\widetilde {W}$ and turbulent transport $\overline {\rho \mathbf {u}^{\prime \prime }W^{\prime \prime }}$ terms on the RHS of Eq. 5, rather than each term separately.

It is also worth noting that the three terms on the LHS of Eq. 5 are similar to the unsteady, mean convection, and turbulent transport terms on the LHSs of the transport equations for $\tilde {\chi } $ [6, 8] or $\bar {\Sigma } $ [3, 4, 9–11], but the RHSs of the latter two equations involve several unclosed terms, contrary to a single unclosed term on the RHS of Eq. 5. This single term (i) differs substantially from any term on the RHS of a transport equation for $\tilde {\chi } $ [6, 8] or $\bar {\Sigma } $ [3, 4, 9–11] and (ii) offers an opportunity to attain a new insight into flame-turbulence interaction, as discussed in detail elsewhere [15, 16].

In the next section, several simple closure relations for the entire convection flux $\overline {\rho \mathbf {u}W}$ will be suggested. In the third section, the attributes of DNS data will be reported. Assessment of the aforementioned closure relations using the DNS data will be discussed in the fourth section, followed by conclusions.

## Simple Closure Relations

Utilizing analogy with quantities $\langle q \rangle _{f}\equiv \overline {q{\Sigma }} / \bar {\Sigma } $ conditioned to flamelets within a mean flame brush [3, 4], let us consider $\langle q \rangle _{r}\equiv \overline {q\rho W} / \overline {\rho W}$ to be a value of a quantity *q*, conditioned to reaction zones. Then, in the statistically planar case addressed in the present paper, a closure relation for the term $\overline {\rho {u}W}=\overline {\rho W}\langle u \rangle _{r}$ is required. Here, *u* is the *x*-component of the flow velocity vector **u** and the *x*-axis is normal to the mean flame brush.

Let us study whether or not the conditioned velocity 〈*u*〉_*r*_ can be evaluated invoking models proposed to determine the conditioned velocity 〈*u*〉_*f*_. It is worth noting that modeling of the mean rate $\overline {\rho W}$ is beyond the scope of the present paper and this quantity will be extracted from the DNS data in the following. The reader interested in modeling $\overline {\rho W}$ is referred to [1–8] and references quoted therein.

As a starting point, let us invoke the following simple relation

6 $$ \langle u \rangle_{f}=\langle c\rangle \overline{u}_{u}+\sigma^{-1}\left( 1-\langle c \rangle \right)\overline{u}_{b}, $$

where *σ* = *ρ*_*u*_/*ρ*_*b*_ is the density ratio, $\overline {u}_{u}$ and $\overline {u}_{b}$ are velocities conditioned to unburned and burned mixture, respectively, and 〈*c*〉 designates either the Reynolds-averaged, $\overline {c}$, or the Favre-averaged, $\tilde {c}$, combustion progress variable, as will be discussed later.

Equation 6 was earlier proposed to model the conditioned velocity 〈*u*〉_*f*_ [17]. This simple model is based on a paradigm of infinitely thin flamelets and on the following reasoning. First, if flamelets are infinitely thin, events associated with arrival of a piece of a flamelet to the trailing edge of a mean flame brush should be accompanied by arrival of unburned gas to the same volume and vice versa. Therefore, $\langle u \rangle _{f}\to \overline {u}_{u}$ at $\overline {c}\to 1$, at least if the density is constant. Second, based on similar arguments, we arrive at $\langle u \rangle _{f}\to \overline {u}_{b}$ at $\overline {c}\to 0$ if *σ* = 1. Thus, in such a case, Eq. 6 is nothing more than a linear interpolation between two limiting conditions. Nevertheless, this simple model is well supported by the results of a recent DNS study of self-propagation of an infinitely thin interface in constant-density turbulence [18].

If flamelet thickness does not vanish and *σ* > 1, we may expect that the aforementioned limit ($\overline {c}\to 1$ or $\overline {c}\to 0$) relations are approximate in the best case, while $\left | \langle u \rangle _{f} \right |>\left | \overline {u}_{u} \right |$ at $\overline {c}\to 1$ and $\left | \langle u \rangle _{f} \right |<\left | \overline {u}_{b} \right |$ at $\overline {c}\to 0$ due to an increase in flow velocity from the unburned to the burned sides of flamelets. Equation 6 addresses such effects in part by multiplying $\overline {u}_{b}$ with *σ* ^−1^. This modification offers an opportunity to allow for the influence of thermal expansion on the limit ($\overline {c}\to 0)$ relation between $\overline {u}_{b}$ and 〈*u*〉_*f*,*u*_ conditioned to the leading edge of flamelets, but $\left | \langle u \rangle _{f,u} \right |<\left | \langle u \rangle _{f} \right |$ due to the aforementioned thermal expansion effects. Accordingly, Eq. 6 exhibits worse agreement with DNS data obtained from flames characterized by *σ* > 1 [19, 20] when compared to the DNS data obtained in the case of *σ* = 1 [18].

It is also worth noting that, first, Eq. 6 is a linear interpolation between two limit points, and thus both $\langle c\rangle =\overline {c}$ and $\langle c \rangle =\tilde {c}$ may be used for the interpolation. Second, in Eq. 6, 〈*c*〉 and $\left (1-\langle c \rangle \right )$ may be substituted with bridging functions $f\left (\langle c \rangle \right )$ and $g\left (\langle c \rangle \right )$ such that $f\left (\langle c \rangle \to 0 \right )\to 0$ and $f\left (\langle c \rangle \to 1 \right )\to 1$ whereas $g\left (\langle c \rangle \to 0 \right )\to 1$ and $g\left (\langle c \rangle \to 1 \right )\to 0$. Third, the conditioned velocities $\overline {u}_{u}$ and $\overline {u}_{b}$ may be multiplied with factors $b_{u}\left (\sigma \right )\ge 1$ and $b_{b}\left (\sigma \right )\le 1$ that allow for $\left | \langle u \rangle _{f} \right |>\left | \overline {u}_{u} \right |$ at $\overline {c}\to 1$ and $\left | \langle u \rangle _{f} \right |<\left | \overline {u}_{b} \right |$ at $\overline {c}\to 0$ due to the influence of thermal expansion on velocity within flamelets. Thus, Eq. 6 may be generalized as follows

7 $$ \langle u \rangle_{r}=f\left( \langle c \rangle \right)b_{u}\overline{u}_{u}+g\left( \langle c \rangle \right)b_{b}\overline{u}_{b}. $$

For instance, because a reaction zone is substantially thinner than a flamelet that contains the zone [7, 21], variations in the flow velocity within the zone along the normal to it could be neglected to the leading order. If the variations in the local flamelet structure are neglected, then one obtains $\rho _{u}\left (\mathrm {\mathbf {u\cdot n}} \right )_{u}=\rho _{r}\left (\mathrm {\mathbf {u\cdot n}} \right )_{r}=\rho _{b}\left (\mathrm {\mathbf {u\cdot n}} \right )_{b}$ due to the local mass conservation. Consequently, *b*_*u*_ = *ρ*_*u*_/*ρ*_*r*_ and *b*_*b*_ = *ρ*_*b*_/*ρ*_*r*_. Here, *ρ*_*r*_ is the density within the reaction zone, e.g. the density conditioned to the peak value of *ρ* *W*.

Strictly speaking, even if $f\left (\langle c \rangle \right ), g\left (\langle c \rangle \right ), b_{u}$, and *b*_*b*_ are known, Eq. 7 does not solve the problem of modeling the velocity 〈*u*〉_*r*_, because the conditioned velocities $\overline {u}_{u}$ and $\overline {u}_{b}$ also require closure relations. However, at least under conditions associated with the flamelet regime of premixed turbulent combustion, the two velocities may be evaluated (i) using the following well-known Bray-Moss-Libby (BML) equations [22, 23]

8 $$ \tilde{\mathbf{u}}=\left( 1-\tilde{c} \right)\overline{\mathbf{u}}_{u}+\tilde{c}\overline{\mathbf{u}}_{b},\qquad \overline{\rho \mathbf{u}^{\prime\prime}c^{\prime\prime}}=\bar{\rho} \tilde{c}\left( 1-\tilde{c} \right)\left( \overline{\mathbf{u}}_{b}-\overline{\mathbf{u}}_{u} \right), $$

(ii) solving the Favre-averaged Navier-Stokes equations in order to determine $\tilde {\mathbf {u}}$, and (iii) invoking some of available models of the flux $\overline {\rho \mathbf {u}^{\prime \prime }c^{\prime \prime }}$, which are reviewed elsewhere [24, 25]. Moreover, models for $\overline {\mathbf {u}}_{u}$ have also been developed, e.g. [26–28]. Accordingly, the conditioned velocities $\overline {u}_{u}$ and $\overline {u}_{b}$ are considered to be known in the present paper and are extracted from the DNS data.

Because turbulent scalar fluxes are often modeled invoking an assumption of gradient diffusion (e.g., to the best of the present authors’ knowledge, solely such models have yet been applied to terms $\overline {\rho \mathbf {u}^{\prime \prime }{\Sigma }^{\prime \prime }}$ and $\overline {\rho \mathbf {u}^{\prime \prime }\chi ^{\prime \prime }}$ in transport equations for mean flame surface density $\bar {\Sigma } $ and mean scalar dissipation rate $\tilde {\chi } $, respectively, [3, 4, 6]), the following closure relations

9 $$ \overline{\rho \mathbf{u}W}=\bar{\rho} \tilde{\mathbf{u}}\widetilde{W}-\bar{\rho} D_{t}{\nabla} \cdot \langle W \rangle , \qquad \overline{\rho \mathbf{u}W}=\bar{\rho} \tilde{\mathbf{u}}\widetilde{W}-D_{t}{\nabla} \cdot \left( \bar{\rho} \widetilde{W} \right) $$

were also tested. Here, 〈*W*〉 designates either $\overline {W}$or $\widetilde {W}$, $D_{t}=C_{\mu } \tilde {k}^{2} / \tilde {\varepsilon } $ is turbulent diffusivity, *C*_*μ*_ is a constant, $\tilde {k}=\overline {\rho \mathbf {u}^{\prime \prime }\cdot \mathbf {u}^{\prime \prime }} / {2\bar {\rho } }$ and $\tilde {\varepsilon } =2\overline {\rho \nu S_{ij}S_{ij}} / \bar {\rho } $ are the Favre-averaged turbulent kinetic energy and its dissipation rate, respectively, which were extracted from the DNS, *ν* is the kinematic viscosity of the mixture, $S_{ij}=0.5\left ({\partial u_{i}} / {\partial x_{j}}+{\partial u_{j}} / {\partial x_{i}} \right )$ is the rate-of-strain tensor, *u*_*i*_ is the *i*-th component of the velocity vector **u**, and summation convention applies to repeated indexes *i* and *j*.

## DNS Attributes

In order to assess closure relations given by Eq. 7 or 9, we analyzed DNS data obtained earlier in two sets of simulations, which were consistent with the framework of the present study (adiabatic burning, low Mach number, single-step chemistry, unity Lewis number). One DNS database (flames H, M, and L) was created by Nishiki et al. [29, 30] by simulating weakly turbulent combustion in the flamelet regime and was analyzed in a number of recent papers [13–16, 27, 31–38]. Another DNS database (flames B, C, D, and E) was created by Chakraborty et al. [39, 40] by simulating combustion in small-scale intense turbulence (the thin-reaction-zone regime [1] of premixed burning) and was also analyzed in a number of recent papers cited elsewhere [15, 16, 27, 41]. Because the DNS attributes were already discussed in detail in the literature, we will restrict ourselves to a very brief summary of the simulations.

In both sets of DNS studies, unsteady 3D balance equations for mass, momentum, energy, and mass fraction of the deficient reactant were numerically solved and the ideal gas state equation was used. Combustion chemical mechanism was simplified by a single Arrhenius type irreversible chemical reaction. The Lewis and Prandtl numbers were taken to be equal to 1.0 and 0.7, respectively. Other basic flame characteristics are reported in Table 1, where *R* *e*_*t*_ = *u* ^′^ *L*/*ν*_*u*_, ${Ka}_{th}=\left ({u^{\prime }} / S_{L} \right )^{3 / 2}/ \left (L / \delta _{th} \right )^{1 / 2}$, *D* *a*_*th*_ = *τ*_*t*_/*τ*_*f*_ are the Reynolds, Karlovitz, and Damköhler numbers, respectively, $ \delta _{th}=\left (T_{b}-T_{u} \right ) / {\max \left | {\nabla } T \right |}$ and *τ*_*f*_ = *δ*_*th*_/*S*_*L*_ are the thermal laminar flame thickness and time scale, respectively, *u* ^′^, *L*, and *τ*_*t*_ = *L*/*u* ^′^ are the rms turbulent velocity, integral length scale of the turbulence, and eddy-turn-over time, respectively.

**Table 1:** Attributes of the cases analyzed here

Case	*σ*	*D* *a*_*th*_	*K* *a*_*th*_	*R* *e*_*t*_	*u* ^′^/*S*_*L*_	*L*/*δ*_*th*_
H	7.53	18.0	0.21	96	0.9	15.9
M	5.0	17.8	0.24	96	1.0	18.0
L	2.5	17.3	0.30	96	1.3	21.8
B	5.5	0.23	13.0	24	6.3	1.4
C	5.5	0.33	13.0	48	7.5	2.5
D	5.5	0.48	13.0	100	9.0	4.3
E	5.5	0.33	19.5	110	11.3	3.75

The computational domains were rectangular boxes of sizes of 8 × 4 × 4 mm or 36.2*δ*_*th*_ × 24.1*δ*_*th*_ × 24.1*δ*_*th*_ in cases H, M, L and B-E, respectively. The domains were resolved using uniform Cartesian meshes of 512 × 128 × 128 and 345 × 230 × 230 cells, respectively. The mean flow velocity was parallel to the *x*-axis and normal to the mean flame brush, with the periodic boundary conditions being set at the transverse sides. Homogeneous isotropic turbulence was used to initialize velocity fluctuations and a single planar laminar flame was embedded into the computational domain at *t* = 0.

In cases B-E, the turbulence decayed with time (reported in Table 1 are the turbulence characteristics at *t* = 0) and averaging was performed over transverse planes at *t*/*τ*_*t*_ = 2 (cases D), 3 (cases C and E), or 4.34 (case B), which amount to one chemical time scale *τ*_*f*_. At those instants, the values of *u* ^′^/*S*_*L*_ decayed by 61% (case B), 45% (case C), 24% (case D), and 34% (case E) in comparison to the initial values reported in Table 1.

In cases H, M, and L, homogeneous isotropic turbulence was generated in a separate box, was injected into the computational domain at *x* = 0, and decayed along the direction *x* (reported in Table 1 are the turbulence characteristics at the inlet). At the leading edges of the mean flame brushes, which were located about 1 mm downstream of the inlet in flames H, M, and L, turbulent kinetic energy was decreased by a factor of about two or three in case H or L, respectively. Accordingly, the Damköhler numbers evaluated at the leading edges were larger than *D* *a*_*th*_ reported in Table 1 by a factor of about 1.5. In case L, the turbulence decay was more pronounced, because the inlet bulk flow velocity was lower (0.8 m/s) than in case H (1.15 m/s). The point is that, in each case, the bulk velocity was equal to the mean turbulent flame speed in order to retain the flame brush within the computational domain for a sufficiently long time [29, 30]. In spite of the turbulence decay, the computed mean turbulent flame speeds were higher than *S*_*L*_ by a factor of about two in cases H, M, and L [29, 30].

After a transition time, which was longer than 1.5*τ*_*t*_ and much longer than *τ*_*f*_, both turbulent burning velocity $U_{T}=\rho _{u}^{-1}{\int }_{0}^{{\Lambda } } {\bar {\rho } \widetilde {W}dx} $ and mean flame brush thickness $\delta _{T}=1 / {\max \left | {\nabla } \overline {c} \right |}$, evaluated in cases H, M, and L, oscillated around statistically steady values [38]. Accordingly, averaging of various quantities $q\left (\mathrm {\mathbf {x}},t \right )$ was performed over transverse planes and over time after the transition period and during the statistically steady period, which was longer than *τ*_*t*_ and much longer than *τ*_*f*_. Subsequently, the obtained dependencies of $\overline {q}\left (x \right )$ were transformed to dependencies of $\overline {q}\left (\overline {c} \right )$ exploiting the monotonicity of the computed axial profiles $\overline {c}\left (x \right )$ of the Reynolds-averaged combustion progress variable.

All quantities reported in the rest of the paper are normalized using *ρ*_*u*_ if applicable, i.e. *ρ* will designate density normalized using the unburned gas density and *ρ*_*u*_ = 1 in the following.

## Results and Discussion

### Weakly turbulent flames H, M, and L

Let us begin with discussing data obtained from flames H, M, and L, associated with the flamelet regime of premixed turbulent combustion. It is worth noting that these data are analyzed in the coordinate framework attached to the mean flame brush such that ${d\overline {c}} / {dx}\ge 0$.

**Fig. 1:**
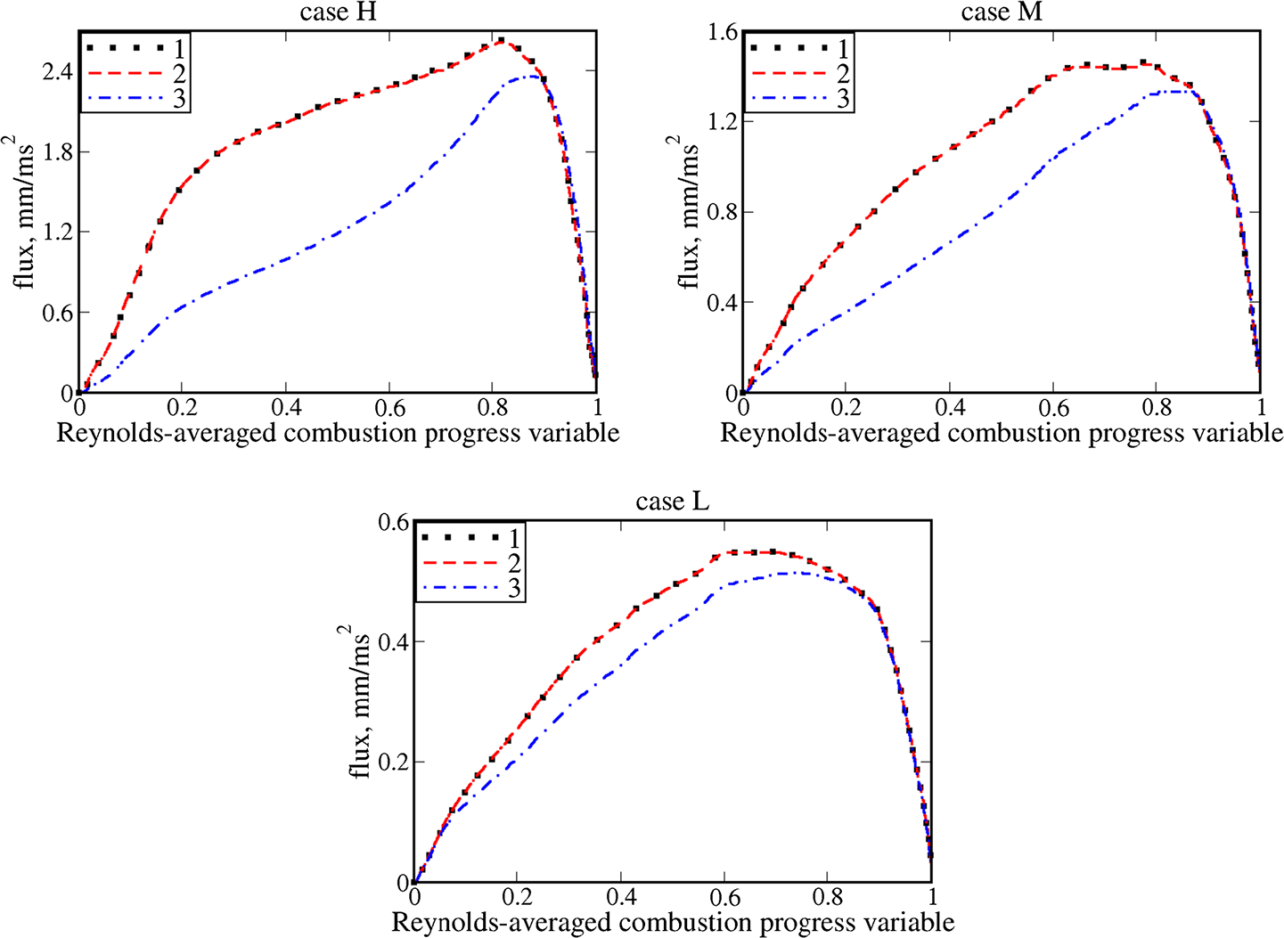
Convection flux vs. Reynolds-averaged combustion progress variable. 1 – $\overline {\rho {u}W}$, 2 – $\bar {\rho } \left \langle {u} |{c_{1}<c<c_{2}} \right \rangle \widetilde {W}$, 3 – $\bar {\rho } \tilde {u}\widetilde {W}$

Figure 1 shows that the conditioned velocity $\langle u \rangle _{r}=\overline {\rho {u}W}/\overline {\rho W}$ introduced above is indeed very close to the velocity conditioned straightforwardly to the reaction zone determined with the following constraint $\rho W>{\max \left \{ \rho W \right \}} / 2$, cf. black symbols and red dashed lines.

Second, at $\overline {c}>0.8$, the entire $\overline {\rho {u}W}$ and mean $\bar {\rho } \tilde {u}\widetilde {W}$ fluxes are very close to one another, thus, indicating that the turbulent flux $\overline {\rho u^{\prime \prime }W^{\prime \prime }}$ is negligible when compared to $\bar {\rho } \tilde {u}\widetilde {W}$ under conditions of the DNS cases considered here.

**Fig. 2:**
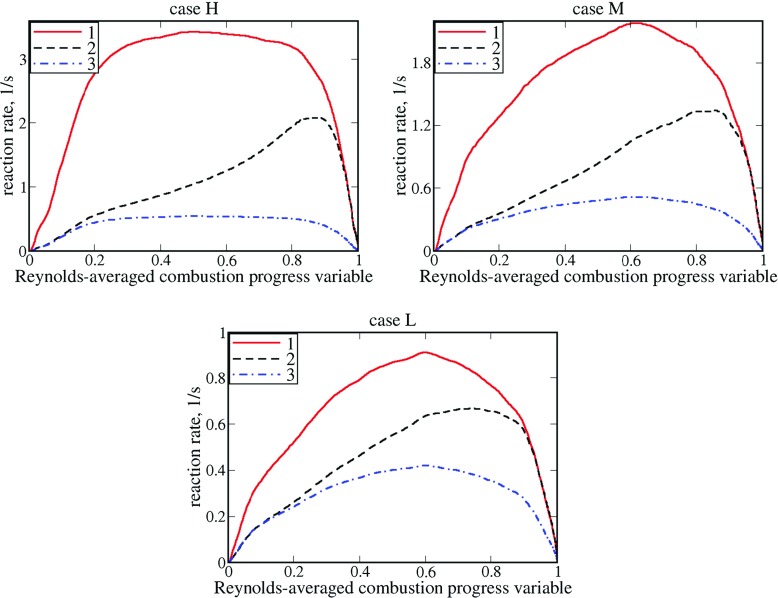
Differently averaged reaction rates vs. Reynolds-averaged combustion progress variable. 1 – $\overline {W}$, 2 – $\widetilde {W}$, 3 – $\overline {\rho W}$

Third, the entire flux $\overline {\rho {u}W}=\bar {\rho } \tilde {u}\widetilde {W}+\overline {\rho u^{\prime \prime }W^{\prime \prime }}$ is significantly larger than the mean flux $\bar {\rho } \tilde {u}\widetilde {W}$ at $\overline {c}<0.8$, cf. black symbols and blue dotted-dashed lines, with the difference being increased by *σ*, cf. cases H, M, and L. Therefore, $\overline {\rho u^{\prime \prime }W^{\prime \prime }}\left (\overline {c}<0.8 \right )>0$, whereas Eq. 9 with *D*_*t*_ > 0 yields the opposite sign of $\overline {\rho {u}W}-\overline {\rho }\tilde {u}\widetilde {W}$ at $\overline {c}<0.5$ in case H or $\overline {c}<0.6$ in cases M and L. Indeed, in these parts of the mean flame brush, both ${d\langle W \rangle } / d\,\overline {c}$ and $ {d\overline {\rho W}} / d\,\overline {c}$ are positive, see Fig. 2, and, hence, both ${d\langle W \rangle } / dx=\left ({d\langle W \rangle } / d\,\overline {c} \right )\left ({d\overline {c}} / {dx} \right )>0$ and ${d\overline {\rho W}} / dx=\left ({d\overline {\rho W}} / d\,\overline {c} \right )\left ({d\overline {c}} / {dx} \right )>0$. The fact that the gradient diffusion closure of the turbulent flux $\overline {\rho u^{\prime \prime }W^{\prime \prime }}$ yields a wrong direction of this flux in flames L, M, and, especially, H is expected, because the flux $\overline {\rho u^{\prime \prime }c^{\prime \prime }}$ shows countergradient behavior in these weakly turbulent flames [30].

As the gradient-diffusion closure given by Eq. 9 is contradicted by the present DNS data at $\overline {c}<0.5$, let us test Eq. 7. For this purpose various bridging functions $f\left (\langle c \rangle \right )$ and $g\left (\langle c \rangle \right )$ and various factors *b*_*u*_ and *b*_*b*_ were invoked. In particular, (i) $f=\overline {c}$, (ii) $f=\tilde {c}$, (iii) $f=\sqrt {\tilde {c}\overline {c}} $, (iv) $f=\sqrt {\tilde {c}} $, (v) $f=\sqrt {\overline {c}} $, (vi) $f=0.5\left (\overline {c}+\tilde {c} \right )$, with *g* = 1 − *f* in all these cases. Moreover, (vii) $f=\overline {c}, g=1-\tilde {c}$ and (viii) $f=\tilde {c}, g=1-\overline {c} $ were also assessed. As far as the factors *b*_*u*_ and *b*_*b*_ are concerned, the following three combinations were tested; 1) *b*_*u*_ = *b*_*b*_ = 1, 2) *b*_*u*_ = *ρ*_*u*_/*ρ*_*r*_ and *b*_*b*_ = *ρ*_*b*_/*ρ*_*r*_, 3) *b*_*u*_ = 1 and *b*_*b*_ = *ρ*_*b*_/*ρ*_*r*_. In the following, we will restrict ourselves to discussing a few closure relations that yield the best agreement with the DNS data on the entire flux $\overline {\rho {u}W}$.

**Fig. 3:**
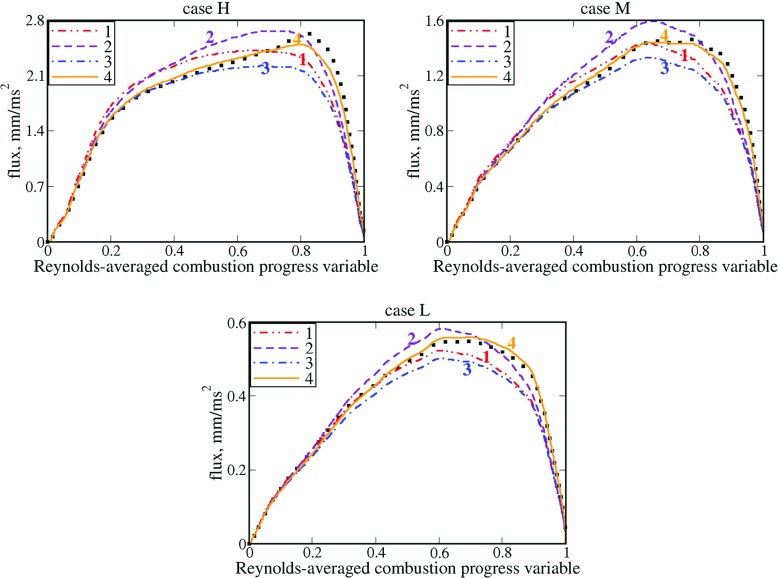
Assessment of various simple closure relations for the entire flux $\overline {\rho {u}W}$. Symbols show DNS data on $\overline {\rho {u}W}$. The same flux $\overline {\rho {u}W}=\overline {\rho W}\langle u \rangle _{r}$ obtained invoking four simple closure relations and DNS data on $\tilde {c}, \overline {c}, \overline {\rho W}, \overline {u}_{u}$, and $\overline {u}_{b}$ is shown in lines. 1 – $\langle u \rangle _{r}=\tilde {c}\overline {u}_{u}+\left (1-\tilde {c} \right )\overline {u}_{b}$, 2 – $\langle u \rangle _{r}=\overline {c} \overline {u}_{u}+\left (1-\tilde {c} \right )\left (\rho _{b} / \rho _{r} \right )\overline {u}_{b}$, 3 – $\langle u \rangle _{r}=\tilde {c}\overline {u}_{u}+\left (1-\tilde {c} \right )\left (\rho _{b} / \rho _{r} \right )\overline {u}_{b}$, 4 – $\langle u \rangle _{r}=\tilde {c}b\overline {u}_{u}+\left (1-\tilde {c} \right )\left (\rho _{b} / \rho _{r} \right )\overline {u}_{b}$, where *b* = 1.2 + 0.04*σ*

Red double-dotted-dashed lines in Fig. 3 show that even the simplest linear interpolation $\langle u \rangle _{r}=\tilde {c}\overline {u}_{u}+\left (1-\tilde {c} \right )\overline {u}_{b}$ yields acceptable agreement with the DNS data. At $\overline {c}<0.5$ in cases M and, especially, H, the agreement is substantially improved by multiplying the conditioned velocity $\overline {u}_{b}$ with a factor of *b*_*b*_ = *ρ*_*b*_/*ρ*_*r*_, see blue dotted-dashed lines, as suggested in the second section. However, the latter model yields slightly worse results at high $\overline {c}$ values. The agreement with the DNS data at large $\overline {c}$ can be improved by substituting $\tilde {c}$ with a larger bridging function $\overline {c}\ge \tilde {c}$ in the first term $\tilde {c}\overline {u}_{u}$ on the RHS in order to increase the magnitude of this term, but such a modification, see violet dashed lines, yields worse results at lower $\overline {c}$.

**Fig. 4:**
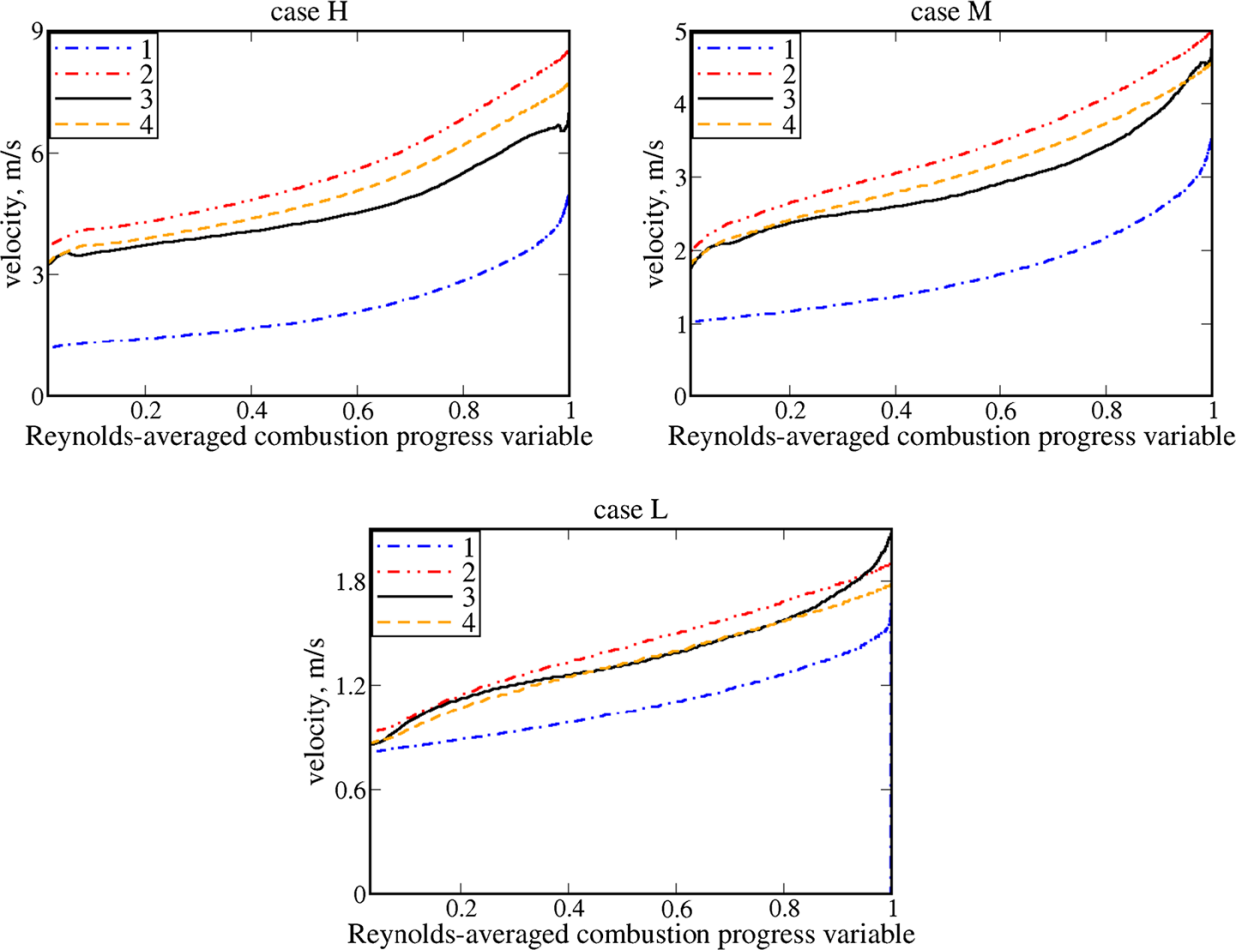
Increase in conditioned velocities within the mean flame brush. 1 – $\overline {u}_{u}$, 2 – $\overline {u}_{b}$, 3 – 〈*u*〉_*r*_ , 4 – ${\rho _{b}\overline {u}_{b}} / \rho _{r}$

A fact that substitution of a factor of *b*_*b*_ = *ρ*_*b*_/*ρ*_*r*_ with unity makes the agreement with the DNS data notably worse at $\overline {c}<0.5$ in cases M and, especially, H, cf. curves 3 and 1, is worth further discussing. On the one hand, in that range of the mean flame brush, 〈*u*〉_*r*_ is mainly controlled by the $\overline {u}_{b}$-term in Eq. 7, whereas the $\overline {u}_{u}$-term plays a minor role, because (i) $\overline {u}_{u}$ is significantly lower than $\overline {u}_{b}$ under conditions of the analyzed DNS, see Fig. 4, and (ii) $\tilde {c}<\left (1-\tilde {c} \right )$ if $\overline {c}<0.5$. Accordingly, a comparison of curves 1 and 3 with the DNS data supports reasoning for introducing a factor of *b*_*b*_ = *ρ*_*b*_/*ρ*_*r*_ into Eq. 7, as discussed in the second section. On the other hand, for the same reasoning, a factor of *b*_*u*_ = *ρ*_*u*_/*ρ*_*r*_ could also be introduced into the $\overline {u}_{u}$-term, but such a model (not shown) significantly overestimates the DNS data, especially at large $\overline {c}$. Therefore, it is worth discussing why 〈*u*〉_*r*_ at $\overline {c}\to 1$ is more close to $\overline {u}_{u}$ than to ${\overline {u}_{u}\rho _{u}} / \rho _{r}$.

In order to clarify the issue, the following feature of premixed turbulent flames appears to be of importance. As discussed in detail elsewhere [38, 42–44], local flamelet structure is strongly perturbed at large $\overline {c}$, where the probability of finding highly curved tips of unburned mixture fingers [38] or cusps [42–44] is substantial. Accordingly a simple relation of $\rho _{u}\left (\mathrm {\mathbf {u\cdot n}} \right )_{u}=\rho _{r}\left (\mathrm {\mathbf {u\cdot n}} \right )_{r}=\rho _{b}\left (\mathrm {\mathbf {u\cdot n}} \right )_{b}$, which is valid in unperturbed flamelets and is invoked to introduce *b*_*u*_ = *ρ*_*u*_/*ρ*_*r*_ and *b*_*b*_ = *ρ*_*b*_/*ρ*_*r*_ in the second section, does not seem to hold near the finger tips or cusps, as the local flamelet curvature is highly negative and the difference in $\overline {u}_{u}$, 〈*u*〉_*r*_, and $\overline {u}_{b}$ is significantly less pronounced at $\overline {c}\to 1$ when compared to the unperturbed laminar flame.

Indeed, Fig. 4 shows that in the trailing part ($\overline {c}>0.9)$ of mean flame brush, the axial velocity of unburned gas is strongly increased, see blue dotted-dashed lines. As discussed elsewhere [38], this increase in $\overline {u}_{u}$ is more pronounced at larger density ratios (case H) and is controlled by the axial pressure gradient induced due to combustion in surrounding flamelets. Consequently, at $\overline {c}\to 1$, the conditioned velocity $\overline {u}_{u}$ reaches values sufficiently close to the mean velocity $\overline {u}$ in products, which is equal to $\overline {u}_{b}\left (\overline {c}\to 1 \right )$. For instance, a ratio of $\overline {u}_{b} / \overline {u}_{u}$ is less than two at $\overline {c}\to 1$ in flame H, whereas the magnitude $\left | \mathrm {\mathbf {u\cdot n}} \right |$ of flow velocity is increased by a factor of 6.5 in the counterpart laminar flame in the coordinate framework attached to it. Thus, at $\overline {c}\to 1$, the local axial acceleration of the flow within flamelets is weakly pronounced and the difference in 〈*u*〉_*r*_ and $\overline {u}_{u}$ is significantly smaller when compared to the laminar flame. Unless a model or theory capable for predicting a ratio of $\langle u \rangle _{r} / \overline {u}_{u}$ at $\overline {c}\to 1$ is developed, the use of the simplest assumption of $\langle u \rangle _{r}\left (\overline {c}\to 1 \right )\to \overline {u}_{u}\left (\overline {c}\to 1 \right )$ appears to be a better choice when compared to invoking an alternative assumption of $\langle u \rangle _{r}\left (\overline {c}\to 1 \right )\to {\overline {u}_{u}\left (\overline {c}\to 1 \right )\rho _{u}} / \rho _{r}$.

Nevertheless, it is worth remembering that the real $\langle u \rangle _{r}\left (\overline {c}\to 1 \right )$ should be between the two limiting expressions (more close to the former one) and, therefore, $\langle u \rangle _{r}\left (\overline {c}\to 1 \right )>\overline {u}_{u}\left (\overline {c}\to 1 \right )$. Substitution of the Favre-averaged $\tilde {c}$ with a larger Reynolds-averaged $\overline {c}$ in the $\overline {u}_{u}$-term in Eq. 7, see curves 2 in Fig. 3, appears to be the simplest way to mimic the difference in $\langle u \rangle _{r}\left (\overline {c}\to 1 \right )$ and $\overline {u}_{u}\left (\overline {c}\to 1 \right )$, but such a modification does not solve the problem, as shown in Fig. 3.

Alternatively, the $\overline {u}_{u}$-term could involve an empirical factor *b*, which is tuned to improve agreement with the DNS data, see orange solid lines in Fig. 3. Such a method allows us to get very good agreement with the DNS data obtained from all three weakly turbulent flames H, M, and L at various $\overline {c}$, with the required tuning of *b* = 1.4 ± 0.1 being more than modest.

Figure 4 shows two more points that are worth noting. First, even at $\overline {c}\to 0$, a ratio of $\overline {u}_{b} / \overline {u}_{u}$ or $\langle u \rangle _{r} / \overline {u}_{u}$ is significantly lower than the counterpart quantity in the unperturbed laminar flame. However, this effect is unlikely to stem from weak local acceleration of the flow in flamelets. Indeed, if we consider a piece of flamelet $x_{f}\left (y,z,t \right )$ that (i) is locally normal to the *x*-axis, i.e. *∂* *x*_*f*_/*∂* *y* = *∂* *x*_*f*_/*∂* *z* = 0, (ii) moves towards the leading edge of the mean flame brush in the coordinate framework attached to it, i.e. *∂* *x*_*f*_/*∂* *t* < 0,and (iii) retain the structure of the unperturbed laminar flame, i.e. the local flow acceleration is as strong as in the laminar flame, then, even in such a case, the axial local flow velocity at the burned side of this element should be less than *σ* *S*_*L*_ (or equal to *σ* *S*_*L*_ if *∂* *x*_*f*_/*∂* *t* = 0). Accordingly, we could assume that $\overline {u}_{b}\left (\overline {c}\to 0 \right )\le \sigma S_{L}$ for the above purely kinematic reasoning. This limit value is equal to 4.5, 2.6, or 1.0 m/s in case H, M, or L respectively, and is sufficiently close to $\overline {u}_{b}\left (\overline {c}\to 0 \right )$ plotted in Fig. 4, see red double-dotted-dashed lines.

Second, comparison of black solid and orange dashed lines in Fig. 4 shows that 〈*u*〉_*r*_ does tend to ${\rho _{b}\overline {u}_{b}} / \rho _{r}$ at $\overline {c}\to 0$, in line with simple reasoning discussed in the second section.

### Thin reaction zone regime flames B-E

Let us consider DNS data obtained from four turbulent flames B-E characterized by low Damköhler numbers and associated with thin reaction zone regime [1] of premixed turbulent combustion. Again, the data are analyzed in the coordinate framework attached to the mean flame brush such that ${d\overline {c}} / {dx}\ge 0$. It is also worth noting that results obtained in those simulations were normalized using the laminar flame speed and thickness.

**Fig. 5:**
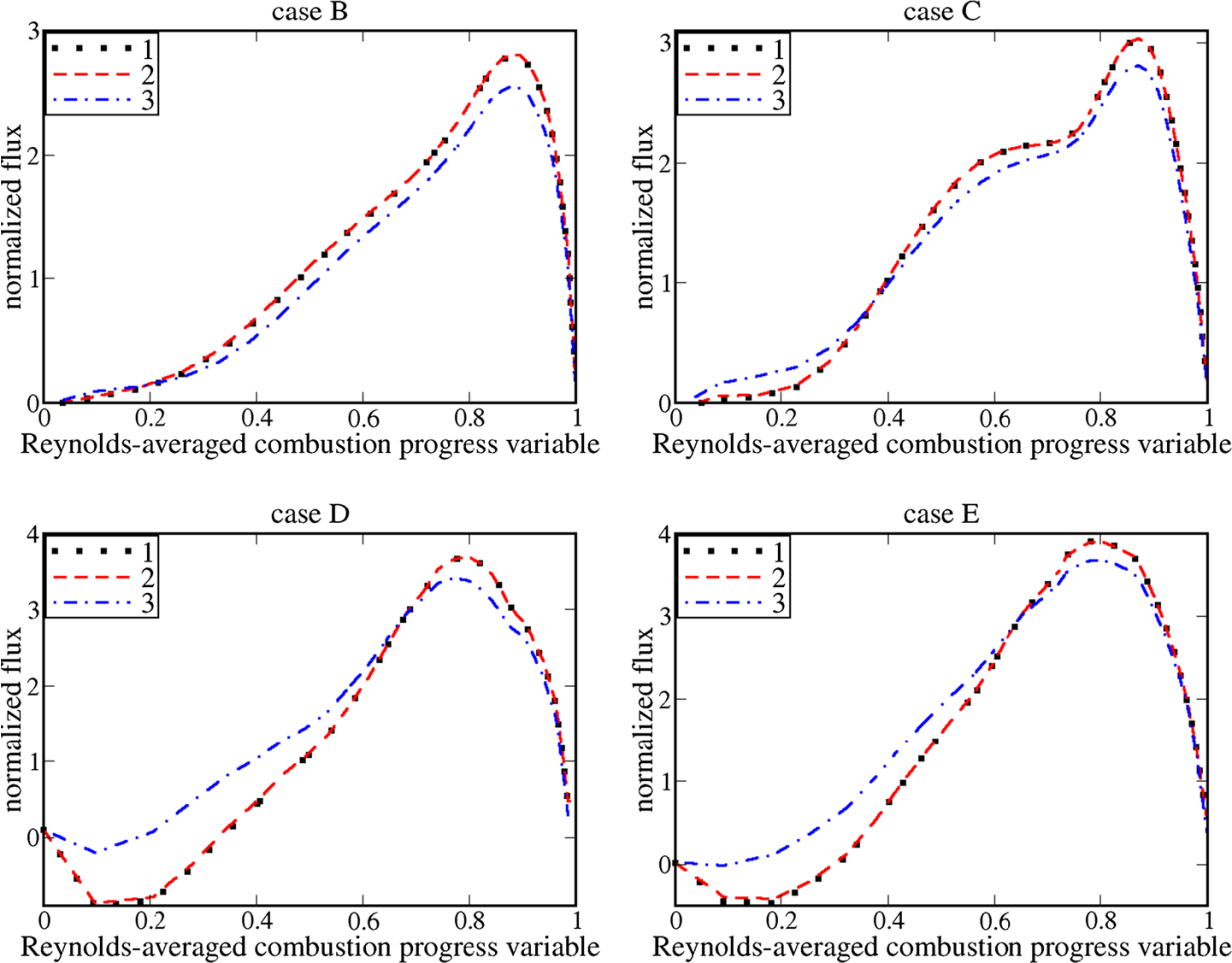
Normalized convection flux vs. Reynolds-averaged combustion progress variable. 1 – $(\delta _{th}/\rho _{u}{S_{L}^{2}})\overline {\rho {u}W}$, 2 – $(\delta _{th}/\rho _{u}{S_{L}^{2}})\bar {\rho } \left \langle {u}| {c_{1}<c<c_{2}} \right \rangle \widetilde {W}$, 3 – $(\delta _{th}/\rho _{u}{S_{L}^{2}})\bar {\rho } \tilde {u}\widetilde {W}$

First, similarly to Fig. 1, Fig. 5 also shows that the conditioned velocity $\langle u \rangle _{r}=\overline {\rho {u}W} / \overline {\rho W}$ is very close to the velocity conditioned straightforwardly to the reaction zone determined with the following constraint $\rho W>{\max \left \{ \rho W \right \}} / 2$, cf. black symbols and red dashed lines, respectively.

Second, similarly to Fig. 1, Fig. 5 also shows that the mean convection flux $\bar {\rho } \tilde {u}\widetilde {W}$ and the entire flux $\overline {\rho {u}W}$ are close to one another in the trailing parts ($0.6<\overline {c})$ of all four mean flame brushes, cf. blue dotted-dashed lines with black symbols, respectively.

Third, at lower $\overline {c}$, the difference in $\bar {\rho } \tilde {u}\widetilde {W}$and $\overline {\rho {u}W}$ is most pronounced in cases D and E, characterized by the highest ratios of *u* ^′^/*S*_*L*_, see Table 1. On the contrary, in cases B and C, the difference between the two fluxes is low in spite of the facts that (i) these two flames are characterized by significantly larger ratios of *u* ^′^/*S*_*L*_ when compared to flames H, M, and L, discussed earlier, but (ii) the difference in $\bar {\rho } \tilde {u}\widetilde {W}$ and $\overline {\rho {u}W}$ is well pronounced in flame M, which is characterized by *σ* = 5.0 close to *σ* = 5.5 in cases B and C. Because flame M is also characterized by a significantly larger ratio of *L*/*δ*_*th*_ when compared to flames B and C, we could assume that the DNS data obtained from all seven flames and considered all together indicate that a role played by the turbulent flux $\overline {\rho u^{\prime \prime }W^{\prime \prime }}$ (i.e. relative magnitude of the difference in $\bar {\rho } \tilde {u}\widetilde {W}$ and $\overline {\rho {u}W})$ is increased by the density ratio (flames H, M, and L), *u* ^′^/*S*_*L*_ (flames B-E), and *L*/*δ*_*th*_ (flames M, B, and C). Definitely, more simulations performed by independently varying *u* ^′^/*S*_*L*_ and *L*/*δ*_*th*_ are required to thoroughly validate such an assumption.

Fourth, in the range of $\overline {c}<0.5$, associated with ${d\langle W \rangle } / dx=\left ({d\langle W \rangle } / d\,\overline {c} \right )\left ({d\,\overline {c}} / {dx} \right )>0$ and ${d\overline {\rho W}} / dx=\left ({d\overline {\rho W}} / d\,\overline {c} \right )\left ({d\overline {c}} / {dx} \right )>0$, the mean convection flux $\bar {\rho } \tilde {u}\widetilde {W}$ is slightly lower (or substantially larger) than the entire flux $\overline {\rho {u}W}$ in flame B (or D and E, respectively), cf. blue dotted-dashed lines with black symbols, respectively. Thus, Fig. 5 indicates transition from a positive (countergradient) turbulent flux $\overline {\rho u^{\prime \prime }W^{\prime \prime }}=\overline {\rho {u}W}-\bar {\rho } \tilde {u}\widetilde {W}$ at $\overline {c}<0.5$ in flame B to a negative (gradient) turbulent flux $\overline {\rho u^{\prime \prime }W^{\prime \prime }}$ at $\overline {c}<0.5$ in flames C, and, especially, D and E. Accordingly, an increase in *u* ^′^/*S*_*L*_, see Table 1, impedes the countergradient turbulent flux $\overline {\rho u^{\prime \prime }W^{\prime \prime }}$, as could be expected via analogy with the turbulent flux $\overline {\rho u^{\prime \prime }c^{\prime \prime }}$, which shows the countergradient (gradient) behavior at lower (larger) values of ${u^{\prime }} / \left (\sigma S_{L} \right )$ [45].

**Fig. 6:**
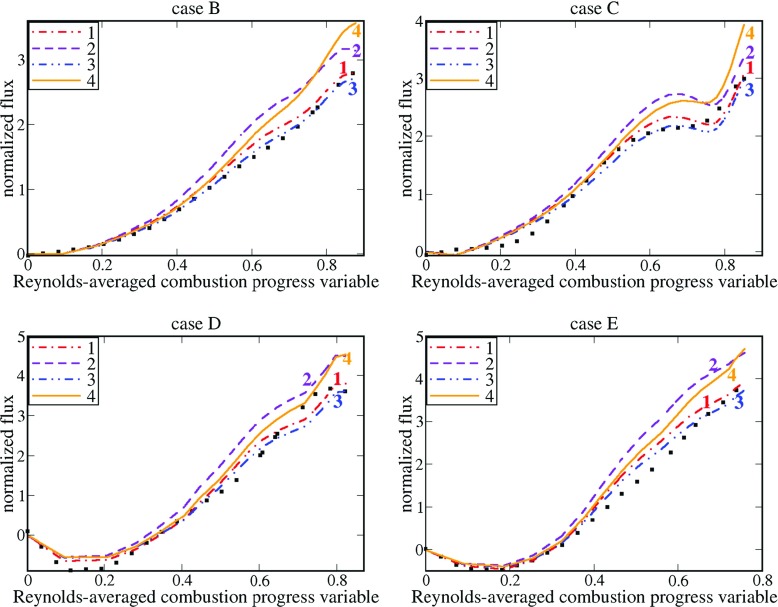
Assessment of various simple closure relations for the entire flux $\overline {\rho {u}W}$. Symbols show DNS data on $(\delta _{th}/\rho _{u}{S_{L}^{2}})\overline {\rho {u}W}$. The same flux $(\delta _{th}/\rho _{u}{S_{L}^{2}})\overline {\rho W}\langle u \rangle _{r}$ obtained invoking four simple closure relations and DNS data on $\tilde {c}, \overline {c}, \overline {\rho W}, \overline {u}_{u}$, and $\overline {u}_{b}$ is shown in lines. 1 – $\langle u \rangle _{r}=\tilde {c}\overline {u}_{u}+\left (1-\tilde {c} \right )\overline {u}_{b}$, 2 – $\langle u \rangle _{r}=\overline {c}\overline {u}_{u}+\left (1-\tilde {c} \right )\left (\rho _{b} / \rho _{r} \right )\overline {u}_{b}$, 3 – $\langle u \rangle _{r}=\tilde {c}\overline {u}_{u}+\left (1-\tilde {c} \right )\left (\rho _{b} / \rho _{r} \right )\overline {u}_{b}$, 4 – $\langle u \rangle _{r}=b\tilde {c}\overline {u}_{u}+\left (1-\tilde {c} \right )\left (\rho _{b} / \rho _{r} \right )\overline {u}_{b}$, where *b* = 1.2 + 0.04*σ* = 1.42

Figure 6 shows that the two simplest expressions, i.e. $\langle u \rangle _{r}=\tilde {c}\overline {u}_{u}+\left (1-\tilde {c} \right )\overline {u}_{b}$ and $\langle u \rangle _{r}=\tilde {c}\overline {u}_{u}+\left (1-\tilde {c} \right )\left (\rho _{b} / \rho _{r} \right )\overline {u}_{b}$ yield very reasonable agreement with the DNS data obtained from all four flames, with the latter equation, see blue double-dotted-dashed lines, doing a little better job when compared to the former one, see red dotted-dashed lines. It is worth noting that Fig. 6 reports DNS data obtained solely in the range of $\overline {c}$ where the number of sample points is sufficient in order to evaluate conditioned quantities in a statistically meaningful manner.

Contrary to flames H, M, and L, discussed earlier, multiplication of $\overline {u}_{u}$ with a factor of *b* = 1 + 0.04*σ* = 1.42 results in a worse agreement with the DNS data at $0.5<\overline {c}$. This difference between results obtained in the two sets of DNS databases could be either attributed to different DNS setups or associated with an eventual decrease in *b* with decreasing Damköhler number. Further target-directed DNSs performed by varying *D* *a*_*th*_ in a wide range of values are definitely required to find the proper explanation.

## Conclusions

The turbulent flux $\overline {\rho u^{\prime \prime }W^{\prime \prime }}$ shows the countergradient behavior in the leading parts ($\overline {c}<0.5)$ of turbulent flame brushes characterized by sufficiently low ratios of *u* ^′^/*S*_*L*_ (cases H, M, L, and B). An increase in *u* ^′^/*S*_*L*_ results in transition from the countergradient to the gradient turbulent flux $\overline {\rho u^{\prime \prime }W^{\prime \prime }}$ (cases D and E) at $\overline {c}<0.5$.

In the trailing parts ($0.8<\overline {c})$ of all seven investigated flame brushes, the magnitude of the turbulent flux $\overline {\rho u^{\prime \prime }W^{\prime \prime }}$ is much smaller than the magnitude of the mean convection flux $\bar {\rho } \tilde {u}\widetilde {W}$.

The following simple closure relation

10 $$ \overline{\rho \mathbf{u}W}=\bar{\rho} \left[ \tilde{c}\overline{\mathbf{u}}_{u}+\left( 1-\tilde{c} \right)\left( \rho_{b} / \rho_{r} \right)\overline{\mathbf{u}}_{b} \right]\widetilde{W}, $$

which does not involve an empirical tuning parameter, offers an opportunity to reasonably well model the entire convection flux $\overline {\rho \mathbf {u}W}$ in the transport equation for the mean reaction rate under substantially different conditions (both weakly turbulent combustion associated with the flamelet regime and burning in small-scale intense turbulence, associated with the thin reaction zone regime).

Under conditions associated with the flamelet regime of premixed turbulent combustion, even better agreement with the DNS data can be obtained by introducing a single empirical parameter *b* = 1.2 + 0.04*σ* = 1.4 ± 0.1 into the following closure relation

11 $$ \overline{\rho \mathbf{u}W}=\bar{\rho} \left[ \tilde{c}b\overline{\mathbf{u}}_{u}+\left( 1-\tilde{c} \right)\left( \rho_{b} / \rho_{r} \right)\overline{\mathbf{u}}_{b} \right]\widetilde{W}, $$

which reduces to Eq. 10 if the tuning constant *b* is skipped. This parameter is required to mimic an increase in flow velocity from the unburned side of a flamelet to its reaction zone due to combustion-induced thermal expansion.

A fact that the value of this model parameter is significantly lower than initially proposed ratio *ρ*_*u*_/*ρ*_*r*_ of the gas densities in the unburned reactants and reaction zone, respectively, is attributed to damping the local flow acceleration due to strong negative curvature of flamelets that are highly probable at the trailing edge of a premixed turbulent flame brush.

## Nomenclature

***b***: Model constant
***c***: Combustion progress variable
***c***_**1**_,***c***_**2**_: Boundaries of the reaction zone
***D***: Molecular diffusivity of a species
***Da***_***th***_ = ***τ***_***t***_/***τ***_***f***_: Damköhler number
***f,g***: Functions
***Ka***_***th***_: Karlovitz number
***k***: Turbulent kinetic energy
***L***: Integral length scale of turbulence
***Le***: Lewis number
${\mathbf {n}}=-{{\boldsymbol {\nabla } }\boldsymbol {c}} / \left | {\boldsymbol {\nabla } }\boldsymbol {c} \right |$: Unit vector locally normal to the flame surface
***q***: Arbitrary quantity
***Re***_***t***_***=******u***^***′***^***L***/***ν***_***u***_: Turbulent Reynolds number
***S***_***d***_: Local displacement speed
***S***_***ij***_: Rate-of-strain tensor
***S***_***L***_: Laminar flame speed
$\dot {\boldsymbol {s}}$: Stretch rate
***T***: Temperature
***t***: Time
***U***_***T***_: Turbulent burning velocity
$\mathbf {u}=\left \{ \boldsymbol {u,v,w} \right \}$: Velocity vector
***u***: Velocity normal to mean flame brush
***u***^′^: rms turbulent velocity
***W***: Rate of product creation
${\boldsymbol {x}}\boldsymbol {=}\left \{ \boldsymbol {x,u,z} \right \}$: Spatial coordinates
***x***: Axis normal to mean flame brush

## *Greek symbols*

$\delta _{\boldsymbol {T}}=1 / {\max \left | {\nabla } \bar {\boldsymbol {c}} \right |} $: Turbulent mean flame brush thickness
$\delta _{th}=\left (\boldsymbol {T}_{b}-\boldsymbol {T}_{\boldsymbol {u}} \right ) / {\max \left | {\nabla } \boldsymbol {T} \right |}$: Laminar flame thickness
*ε*: Rate of dissipation of turbulent kinetic energy
*κ*: Heat diffusivity of a mixture
*ν*: Kinematic viscosity
*ρ*: Density
##Σ##: Flame surface density
*σ* = *ρ*_*u*_/*ρ*_*b*_: Density ratio
*τ*_*f*_ = *δ*_*t**h*_/***S***_***L***_: Laminar flame time scale
*τ*_*t*_ = ***L***/***u***^′^: Turbulent time scale
*χ*: Scalar dissipation rate

## *Subscripts and superscripts*

$\overline {q} $: Reynolds-averaged value of a quantity *q*
$\tilde {q}=\overline {\rho q} / \bar {\rho } $: Favre-averaged value of a quantity *q*
〈*q*〉: Either Reynolds or Favre-averaged value of a quantity *q*
〈*q*|*c*_1_ < *c* < *c*_2_〉: Value of a quantity *q*, conditionally averaged at *c*_1_ < *c* < *c*_2_
$\langle q\rangle _{f} =\overline {q\sum } /\overline \sum ~~$: Value of a quantity *q* conditioned to flamelets
$\langle q \rangle _{r} =\overline {\rho qW} / \overline {\rho W} $: Value of a quantity *q* conditioned to reaction zone
$q^{\prime \prime }=q-\tilde {q}$: Fluctuations with respect to Favre-averaged values
*b*: Burned
*f*: Flamelet
*r*: Reaction zone
*T*: Turbulent
*u*: Unburned
